# New genomic resources to boost research in reproductive biology to enable cost‐effective hybrid seed production

**DOI:** 10.1002/tpg2.70092

**Published:** 2025-08-07

**Authors:** Antje Rohde, Marc C. Albertsen, Scott A. Boden, Pauline Bansept‐Basler, Philipp H. G. Boeven, Colin Cavanagh, Laura E. Dixon, Claus Frohberg, Lucie Griffe, Jacob Lage, Leah Maeder, Marina Millán‐Blánquez, Paul D. Olson, Laura Röhrig, Thorsten Schnurbusch, Cristóbal Uauy, Ryan Whitford

**Affiliations:** ^1^ KWS SAAT SE Einbeck Germany; ^2^ Albertsen Crop Genetics for Humanity LLC Johnston Iowa USA; ^3^ School of Agriculture, Food and Wine University of Adelaide Adelaide South Australia Australia; ^4^ Bayer Seeds SAS, Centre de recherche Boissay Toury France; ^5^ Limagrain GmbH Peine‐Rosenthal Germany; ^6^ BASF Australia Limited Southbank Victoria Australia; ^7^ Leibniz Institute of Plant Genetics and Crop Plant Research (IPK) Gatersleben Germany; ^8^ BASF Innovation Center, BASF Belgium Coordination Center CommV Gent Gent Belgium; ^9^ RAGT Seeds Ltd. Ickleton UK; ^10^ KWS Momont Mons en Pévèle France; ^11^ Corteva Agriscience Johnston Iowa USA; ^12^ Laboratoire Reproduction et Développement des Plantes Univ Lyon, ENS de Lyon, UCB Lyon 1, CNRS, INRAE Lyon France; ^13^ Syngenta Seeds GmbH Bad Salzuflen Germany; ^14^ Martin Luther University Halle‐Wittenberg, Faculty of Natural Sciences III, Institute of Agricultural and Nutritional Sciences Halle Germany; ^15^ John Innes Centre, Norwich Research Park Norwich UK; ^16^ Centre for Crop and Food Innovation, State Agricultural Biotechnology Centre, Food Futures, Institute Murdoch University Murdoch Western Australia Australia

## Abstract

The commercial realization of hybrid wheat (*Triticum aestivum* L.) is a major technological challenge to sustainably increase food production for our growing population in a changing climate. Despite recent advances in cytoplasmic‐ and nuclear‐based pollination control systems, the inefficient outcrossing of wheat's autogamous florets remains a barrier to hybrid seed production. There is a pressing need to investigate wheat floral biology and enhance the likelihood of ovaries being fertilized by airborne pollen so breeders can select and utilize male and female parents for resilient, scalable, and cost‐effective hybrid seed production. Advances in understanding the wheat genomes and pangenome will aid research into the underlying floral organ development and fertility with the aim to stabilize pollination and fertilization under a changing climate. The purpose of this position paper is to highlight priority areas of research to support hybrid wheat development, including (1) structural aspects of florets that affect stigma presentation, longevity, and receptivity to airborne pollen, (2) pollen release dynamics (e.g., anther extrusion and dehiscence), and (3) the effect of heat, drought, irradiation, and humidity on these reproductive traits. A combined approach of increased understanding built on the genomic resources and advanced trait evaluation will deliver to robust measures for key floral characteristics, such that diverse germplasm can be fully exploited to realize the yield improvements and yield stability offered by hybrids.

## Core Ideas

- Wheat pollination in line and hybrid varieties needs to be secured in a changing climate.
- Cost‐effective hybrid wheat seed production requires selection for outcrossing floral organs.
- Wheat breeding companies and researchers are calling for adequate funding for wheat floral biology research.

**Abbreviations**
CHA: chemical hybridizing agent
CMS: cytoplasmic male sterility
EPP: effective pollination period
NMS: nuclear male sterility
QTL: quantitative trait loci

## 1 INTRODUCTION

Bread wheat (*Triticum aestivum* L.) is vital for the global diet, providing grain that accounts for ∼20% of our protein and calories (Erenstein et al., 2022). Breeding and agronomy have more than doubled grain production from the 1960s to 2010 (Fischer et al., 2014). However, a recent stagnation in yield gains (<1% globally) necessitates the adoption of innovative approaches to achieve the estimated 60%–70% productivity increase required by 2050 to sustain global food security (Gerber et al., 2024; Van Dijk et al., 2021). Hybrid breeding has proven exceptionally successful in driving yield increases for crops like rice (*Oryza sativa* L.) and maize (*Zea mays* L.); maize production, for example, has doubled since the 1960s, representing an annual 2% yield improvement (Fischer & Edmeades, 2010). Surveys of experimental wheat hybrids in central Europe and the United States conservatively estimate that 10%–24% yield increases and doubling yield stability are achievable (Easterly et al., 2020; Gowda et al., 2012; Longin et al., 2013; Longin et al., 2015; Longin et al., 2012; Mühleisen et al., 2014; Schneider, Frels, et al., 2024). Despite the potential benefits of hybrid wheat, implementation of this technology has been hampered by the complexity of cross‐pollination, which impedes efficient hybrid seed production. An important note is that the “seed,” we discuss here, is in fact a fruit‐like structure, called caryopsis, and is therefore correctly referred to as grain; however, given industry commonly defines grain for consumption and seed for sowing a crop, we will proceed with using “seed.”

The commercial production and deployment of wheat hybrids demands three prerequisites to be developed: (1) an effective pollination control system that facilitates obligate outcrossing between selected parents, (2) an economically viable production of hybrid seeds at scale, and (3) maximal heterotic yield advantage in the hybrids. Revell et al. (2025) have recently reviewed the options for robust and practicable pollination control, such as chemical hybridizing agents (CHAs; such as Croisor 100, Genesis) and cytoplasmic and nuclear controlled male sterility systems (cytoplasmic male sterility [CMS] and nuclear male sterility [NMS], respectively) (Darvey et al., 2019; Revell et al., 2025; Singh et al., 2021; Singh et al., 2018). Clearly, NMS is a leading system, as it limits the number of cross‐pollination steps in hybrid production, no longer necessitating cross‐pollination for sterile parent maintenance as does CMS, and is also not dependent on specific environmental cues (Revell et al., 2025). While these pollination control systems enable the production of intended hybrids with acceptable heterosis levels of 10%–20% (Gupta et al., 2019; Longin et al., 2013; Schneider, Hinterberger, et al., 2024), wheat's self‐pollinating florets restrict the large‐scale and economically feasible production of hybrid seed (Selva et al., 2020; Whitford et al., 2013).

The economics of hybrid wheat for a breeder are primarily determined by the cost of producing hybrid seed and the expected seeding rate used by the grower. Hybrid seed production, whether for test crosses or commercial production, depends on synchronizing female receptivity with the availability of airborne pollen from a chosen male donor (termed nicking). Ideally, the female parent flowers 2–5 days earlier than the male parent (Schmidt et al., 2024), and the male is taller than the female genotype to enable pollen flow; height can be manipulated through exploitation of *Rht* genes (Würschum et al., 2018), for example. Hybrid seed production that harnesses CMS/NMS pollination control systems is more cost‐effective than CHA‐controlled systems, but all require favorable floral morphologies.

Often, hybrid seed production is done in isolated strip planting with male and (male‐sterile) female parental lines planted next to each other (and isolated from other sources of wheat pollen). Other approaches to improve the affordability of hybrid seed production include blended hybrids (Wilson, 1997), where seeds from a male‐sterile female and a fertile male are mixed, resulting in hybrid seeds that contain a small fraction of inbred males. In such a mixed planting, the distance required for pollen to travel is less than in a strip planting. However, such blended hybrid seed must pass official varietal testing requirements, which vary between countries. A third approach to hybrid seed production involves the application of collected and stored pollen to female acceptor lines at the peak of their receptivity, such as in hybrid rice and maize (Brena et al., 2019; www.powerpollen.com).

**FIGURE 1:**
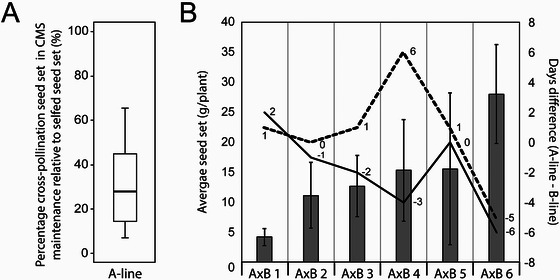
Variation in cross‐pollination seed set in cytoplasmic male sterility (CMS) line maintenance. (A) Meta‐analysis summarizing seed set of a test set of CMS females (A‐lines) as percent of self‐fertile B‐lines seed set from Limagrain 2020–2023 CMS maintenance across German research locations. (B) Seed set of six different CMS (A‐lines) pollinated by the respective near‐isogenic maintainer (B‐line), as observed in RAGT trials. The bar chart represents the average seed set per genotype ± standard deviation. The solid line represents the difference in spike emergence date in the A‐line and the B‐line. The dashed line represents the difference between the start of gaping in the A‐line and the start of flowering in the B‐line.

In the CMS‐based, three‐line pollination control systems, cross‐pollination is not only required to create the F1 hybrids but also for the maintenance and multiplication of the CMS line (A‐line) by an isogenic fertile maintainer (B‐line). The relative seed set in CMS maintenance can vary widely and yields less on average compared to self‐pollination (Figure 1A). In an independent data set, the variation in seed set among six A x B pairs suggested a trend that spike emergence in the CMS line (A‐line) needs to be a few days ahead of the B‐line for better seed set (Figure 1B). However, additional factors are likely to influence seed set in the CMS lines, which could be environmental factors, such as temperature, rain, and wind, and phenotypical factors, such as female receptivity, anther extrusion, pollen quantity and quality. While breeders typically select lines for excellent pollen shed and good seed set after cross‐pollination, during CMS maintenance, earlier gaping of the A‐line and full flowering of the B‐line need to be achieved in a near‐isogenic background (same nuclear background) that limits the genetic options for improving cross‐pollination as well as preventing the utility of mixed planting.

**Core Ideas**

- Wheat pollination in line and hybrid varieties needs to be secured in a changing climate.
- Cost‐effective hybrid wheat seed production requires selection for outcrossing floral organs.
- Wheat breeding companies and researchers are calling for adequate funding for wheat floral biology research.

**FIGURE 2:**
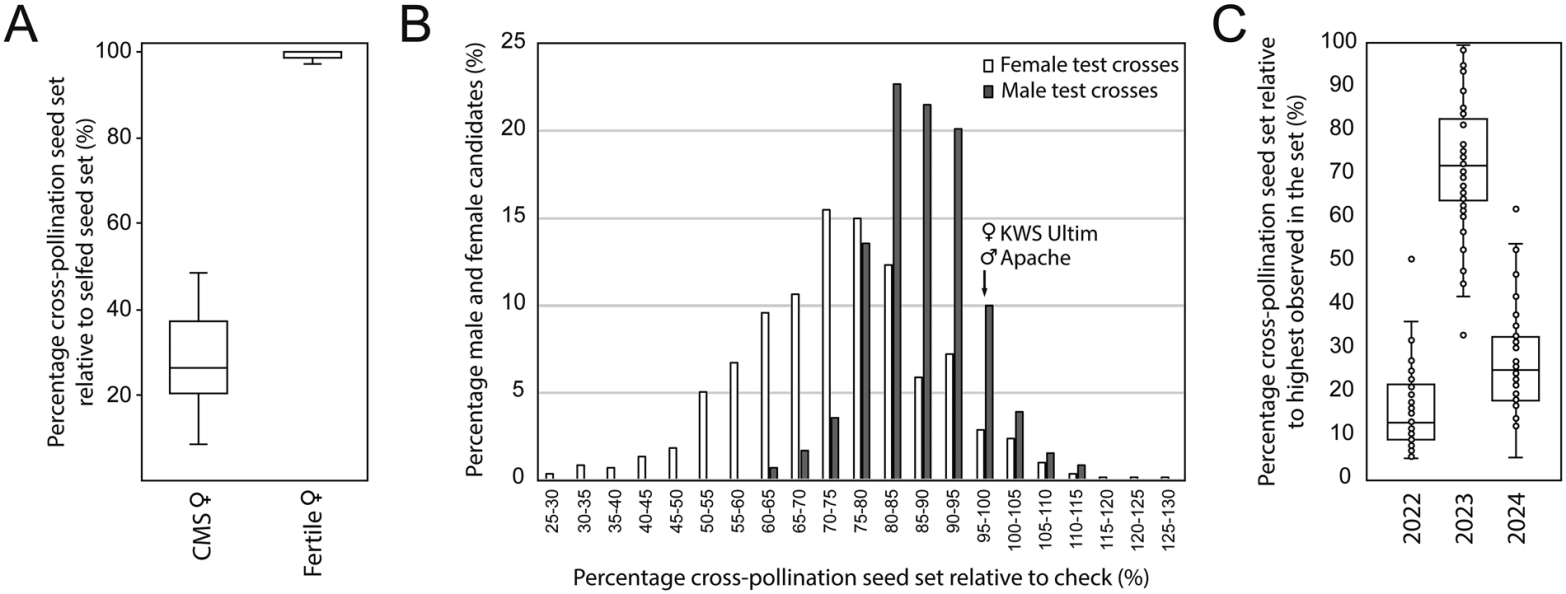
Variation in cross‐pollination seed set in hybrid production. (A) Meta‐analysis summarizing cross‐pollination seed set on cytoplasmic male sterility (CMS) females as percent of self‐fertile female maintainer seed set from Corteva 2022–2024 hybrid productions across North American research locations. (B–C) seed set from top crosses using a chemical hybridizing agent (CHA). (B) Seed set was measured in the breeding program of KWS on a total of 1185 CHA‐treated parental genotypes (594 females, 591 males) after cross‐pollination with a tester from the opposite pool. Cross‐pollination seed set is expressed relative to hybrids involving genotypes KWS Ultim (♀) and Apache (♂). (C) Seed set of 50 hybrid combinations was repeatedly measured in the breeding program of BASF across the years 2022, 2023, and 2024. Cross‐pollination seed set is expressed relative to the hybrid with the highest seed yield.

Hybrid seed production in cross‐pollinating settings is challenging regardless of the pollination control and production system. Again, breeders select lines to be engaged in test cross hybrids or hybrids by ensuring excellent pollen shed in the male pool and good seed set in the female pool. Still, for example, in a large set of CMS females, <50% seed set in hybrid productions was observed across multiple locations and years compared to self‐fertile check genotypes (Figure 2A). This again demonstrates that factors other than nicking, gaping of the CMS line, and anther extrusion in the male seem to influence effective seed set in the female. Also, seed set in CHA‐controlled test crosses ranges from 25% to 130%, relative to seed set in benchmarking hybrids (Figure 2B). Further, seed set strongly varies with environmental conditions encountered during flowering, as shown with 50 identical hybrid production trials across years (Figure 2C).

Furthermore, a recent study on CHA‐controlled hybrid seed yield could only explain one‐third of the genetic variation in hybrid seed yield with plant height and flowering time but failed to identify any other significant marker‐trait associations, suggesting hybrid seed yield is subject to highly polygenic control (Schneider, Hinterberger, et al., 2024).

Together, these data highlight floral architecture and fertility‐related traits as key targets for research and development activities that aim to enhance hybrid seed production. Our alliance of commercial companies, which are committed to hybrid wheat utilizing diverse pollination control systems, agree that cross‐pollination seed set limits the number of candidate hybrid parents available, imposing a restriction on the diversity of germplasm entering advanced stages of breeding programs. This represents a significant improvement opportunity for enabling maximum heterosis and crop performance for farmers as well as reasonable costs of goods to better encourage continued private investment in wheat crop enhancement. We propose that wheat's floral morphology needs to be modified to optimize outcrossing efficacy, with female and male traits, as outlined below. An enhanced understanding of these floral traits and their responses to diverse environmental conditions will likely benefit sustained productivity of hybrid and inbred genotypes in a changing environment, especially in warmer and drier conditions. Encouragingly, recent advances in our understanding of wheat genomes (Jiao et al., 2025; Tiwari et al., 2024) will provide new opportunities to decipher the genetic variation underlying diversity in floral organ traits. For example, under a conservatively estimated rise of 1.5°C in the near future, grain yields are predicted to decrease by 7%–12% (Zaveri & Lobell, 2019). This impact will involve water and heat stress that have already been demonstrated to reduce seed production by 54% in one out of 3 years for hybrid seed set evaluation of 72 winter wheat lines (Schneider, Frels, et al., 2024).

## 2 CURRENT UNDERSTANDING OF FLORAL ORGAN DEVELOPMENT AND POLLINATION

**FIGURE 3:**
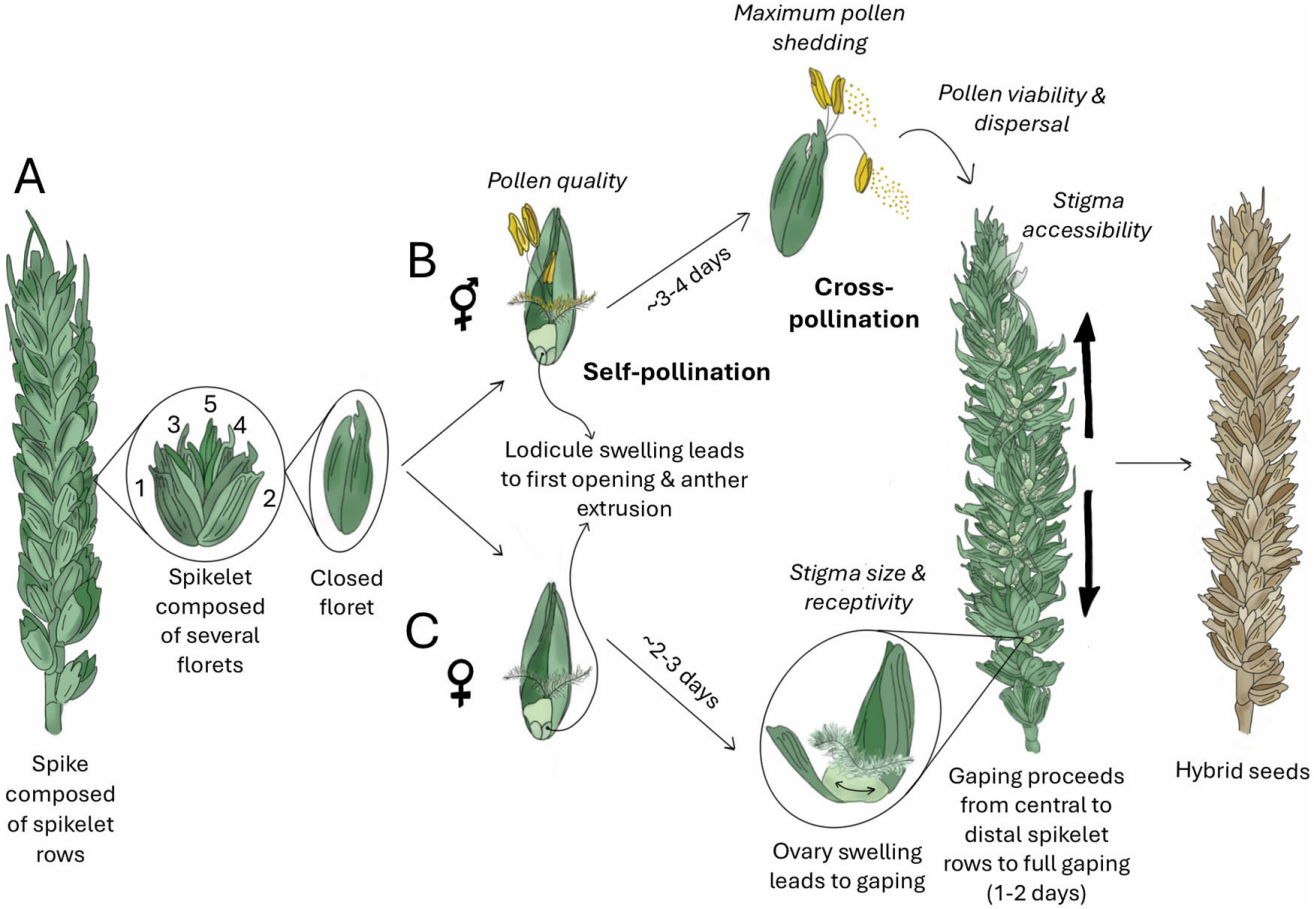
Wheat inflorescence and pollination. The scenario of self‐ and cross‐pollination are illustrated as occurring in hybrid seed production for the male pollinator (B) and the sterile female seed parent (C) (De Vries, 1971; Khan et al., 1973; Kirby, 2002; Okada et al., 2018; Waddington et al., 1983).

The wheat inflorescence is composed of sessile spikelets that are alternately distichously arranged along a central axis, called a rachis (Figure 3A). Each spikelet is enclosed by two bract‐like glumes and contains several florets. Each floret is comprised of two bract‐like protective structures known as a lemma (abaxially) and palea (adaxially), which safeguard a pair of lodicules (homologous to eudicot petals) and the sexual organs, three stamens, and a pistil. The pistil is comprised of three fused carpels surrounding a single ovule. The apical region of the pistil bifurcates into two styles with feathery stigmas. Given this enclosed floret structure, wheat is strongly autogamous. In self‐fertile plants, pollination occurs early inside the opening, yet still closed floret, almost concurrent with anther dehiscence, lodicule swelling, and filament elongation, which ultimately results in anther extrusion if palea and lemma gape (Figure 3B; Zajączkowska et al., 2021). Pollen that is not released in the floret is then shed outside at maximum anther extrusion and dispersed by wind. As a result of these events, self‐fertile wheat cross‐pollinates at a low frequency (Boeven et al., 2016; Würschum et al., 2018; Zajączkowska et al., 2021). In male‐sterile plants, florets first open as the lodicules enlarge, and they continue to a “second opening” through the action of circumferential ovary swelling that only occurs in the absence of fertilization, often described as floret gaping (Figure 3C; Okada et al., 2018; Schmidt et al., 2024). Gaping proceeds from central to distal spikelets (Figure 3C). When successfully cross‐pollinated, hybrid seeds are set on this female parent. Ideal cross‐pollinators should possess high anther extrusion, facilitated by long filaments, large lodicules, and soft pliable paleae, lemmas, and glumes (Whitford et al., 2013). Anthers should produce abundant, long‐lived pollen that is easily dispersed, with inflorescences positioned higher than the female to promote greater wind‐driven pollen dispersal and capture (Denisow et al., 2022; Whitford et al., 2013).

Many floral characteristics, such as anther extrusion, anther length, pollen mass, duration of floret opening, floret openness, and stigma length, are heritable and exhibit genotypic variance (El Hanafi et al., 2020). Pollen mass and anther extrusion were found repeatedly to correlate highly with seed yield (Boeven et al., 2018; El Hanafi et al., 2022; Garst et al., 2023), and similarly, they were also found to correlate with the duration of floret openness (Schmidt et al., 2024).

Our current understanding of the importance that male organs play in hybrid seed set mainly relates to pollen shed as a function of anther extrusion and the associated traits of anther size, pollen number per anther, and collected pollen mass (Langer et al., 2014; Nguyen et al., 2015). Schierenbeck et al. (2024) showed that green revolution wheat varieties (genotypes carrying *Rht* semi‐dwarfing alleles) inherently display poor male floral characteristics (i.e., anther extrusion) (Schierenbeck et al., 2024), which is supported by multiple independent genome‐wide association studies and analyses of biparental mapping populations (Boeven et al., 2016; Buerstmayr & Buerstmayr, 2016; Garst et al., 2023; He, Lillemo, et al., 2016; He, Singh, et al., 2016; Langer et al., 2014; Lu et al., 2013; Muqaddasi et al., 2017; Okada et al., 2019, 2018, 2020; Sade et al., 2022). The proportion of extruded anthers also strongly associates with post‐anthesis filament length (Denisow et al., 2022).

Few studies have implicated the importance of female organs in the efficiency of hybrid seed production. Studies across genotypically diverse (Schneider, Hinterberger, et al., 2024) and small panels of elite females (Boeven et al., 2018; Schneider et al., 2021) have demonstrated that pollen receptivity is critical for hybrid seed set, although no clear links to observable female phenotypes have been drawn. Spikes with a *lax* arrangement of spikelets, however, have been shown to improve floret opening and enhance accessibility of stigmas to airborne pollen, which facilitated cross‐pollination relative to plants with more compact spikelets (Pickett, 1993). The length and structure of the stigma also influence receptivity (Pickett, 1993; Whitford et al., 2013).

Stigmas undergo three stages of development: growth to reach ∼85% of maximum size; peak, lasting until stigma area decreases 15% from maximum; and then deterioration, characterized by gradual senescence (Millán‐Blánquez et al., 2022). The phases of stigma development are guided by morphological cues, and significant transcriptional changes associated with these cues may provide leads for targeted genetic modulation to fine tune different aspects of stigma biology (Millán Blánquez, 2023; Millán‐Blánquez et al., 2022). Independent of being CMS or NMS, different male‐sterile varieties show distinct developmental rates across these phases and form different‐sized stigmas. Stigma longevity limits seed set; however, ensuring pollen can access stigmas is of higher importance for improving hybrid seed production under field conditions (Millán‐Blánquez et al., 2025). The *Rht14* dwarfing locus, derived from durum wheat Italo (*Triticum durum* Desf.), has also been shown to improve stigma presentation in hexaploid bread wheat (Pallotta et al., 2024).

**FIGURE 4:**
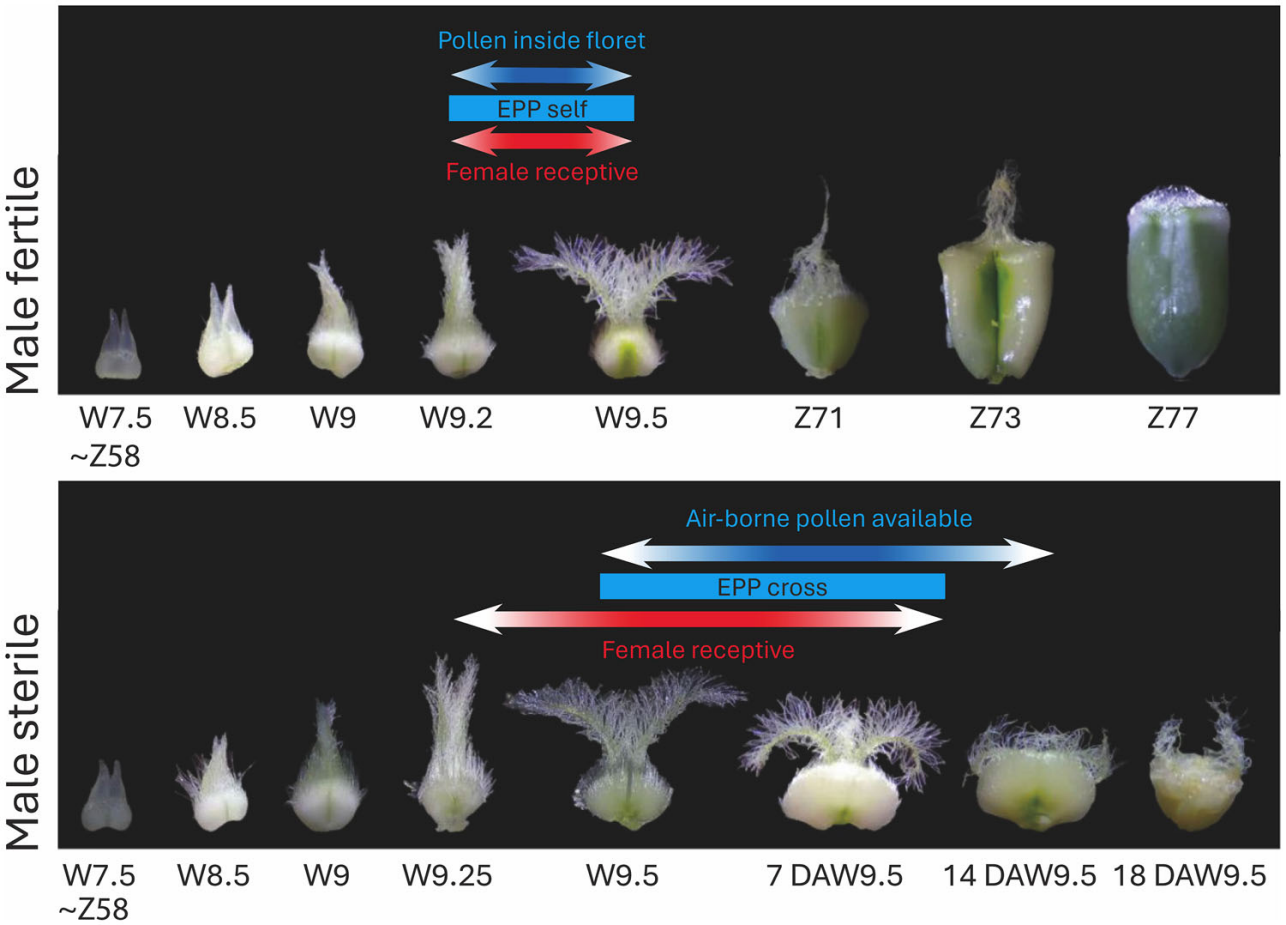
Effective pollination period (EPP). The effective pollination period differs in self‐ and cross‐pollinating scenarios. In selfing, pollen is shed on the receptive stigma within a yet closed floret. In cross‐pollination, pollen needs to be available outside the floret to shed on a male‐sterile stigma and usually coincides with a gaping, receptive female. The dynamics of anther extrusion and pollen shedding, as well as stigma growth, presentation, and receptivity, differ widely across genotypes (Millán‐Blánquez et al., 2022; Millán‐Blánquez et al., 2025; Pallotta et al., 2024). Anther dehiscence (rupture of anther lobes and pollen release) marks the start of the EPP in the male organs, while it stops when all pollen are non‐viable, as can be determined by staining. The female EPP is less well understood, but stigma/styloid protrusion or exposure to outside the palea/lemma in sterile florets marks the onset, with stigma hair atrophication marking the loss of the stigma vitality. A quantitative scale is provided based on previously published developmental scales (W: Waddington; Z: Zadoks; DAW9.5: Days After Waddington 9.5). The last Zadoks stage (Z53), useful for developmental staging in practical breeding settings prior to the first Waddington stage, is indicated tentatively, as this varies across genotypes. Images not to scale. Images adapted from Millán Blánquez (2023).

While studies have addressed male and female organs in isolation, the synchronization and interaction of gametes determine the reproductive success that goes beyond the perfect overlap in flowering (nick). We propose to focus on this interaction, and we introduce the term “effective pollination period” (EPP) to the wheat community (Figure 4). The EPP refers to the phase during which male and female gametes are viable and capable of facilitating gamete fusion and fertilization, which can be enhanced by organ morphology (Sanzol & Herrero, 2001; Williams, 1965). In cross‐pollination, anther extrusion and dehiscence outside the floret need to be synchronized with peak stigma exertion and female receptivity. Male and female development may be or become asynchronous within a floret and certainly across different florets on (female and male) plants in a cross‐pollination setting, each of these being exacerbated under stress such as heat or drought (Lukac et al., 2011). Such male to female asynchrony (within a floret) occurs in the naturally outcrossing cereal rye (*Secale cereale* L.) (Bennett et al., 1973), showing that organ development could potentially shift toward beneficial states in only one organ, for example, later anther dehiscence when cross‐pollination needs to be met.

The synchrony and duration of the EPP are variable under changing weather conditions. Understanding genetic components that reduce this variability is essential for robust control of the EPP to maximize hybrid seed set across environments and seasons. It is well documented that pollen viability decreases under warmer (over 32°C) and drier conditions (De Storme & Geelen, 2014; Fábián et al., 2019; Saini & Aspinall, 1982). However, we also need to understand the processes that trigger pollen release and whether they can be adjusted to occur during cooler parts of the day, maximising pollen fertility; the circadian regulation of floral organ development has been reported in other species (Marshall et al., 2023). The environment also impacts other phases of reproductive development that will influence effective pollination; for example, temperature and day length affect early spikelet and floret development, and flowering time is delayed when the cooler temperatures of winter and early spring, needed for vernalization and flowering in winter cultivars, are interrupted with a higher temperature (Dixon et al., 2019). Understanding the changes impacting EPP, together with the environmental impact on stem elongation and floral emergence, will help breeders optimize cross‐pollination seed set and therefore production efficiencies.

## 3 RESEARCH NEEDED FROM A BREEDER'S PERSPECTIVE

1. *Effective phenotyping of female receptivity*: Research is needed to describe female reproductive organ development, including the alignment of gamete development and the interaction of female and male organs throughout pollination and fertilization. A comprehensive understanding will extend the current Waddington scale and Waddington+ scales (Schmidt et al., 2024; Waddington et al., 1983) and establish a common framework, scale, and terminology for key molecular and biological events that underpin fertility. The fundamental understanding should aid development of a much‐needed practical phenotyping method for receptivity that can exploit diverse genetic resources. An example includes linking the receptivity from fine‐scale experimentation to macroscopically observable changes, such as glume flaring or peduncle length.
2. *Stigma receptivity*: Breeders indirectly select for receptivity through seed set by cross‐pollination, but this assay is biased by their choice of a tester genotype, agronomic practice, and climatic conditions. More direct targets for selection of favorable stigma characteristics are desirable; however, quantitative trait loci (QTL) for stigma receptivity or floret openness have yet to be identified in wheat. In rice, stigma presentation (exertion) outside the lemma and palea is a significant contributor to airborne pollen capture and hybrid seed production yields (Xie, 2009; Zhou et al., 2017), with outcrossing rates largely correlating with stigma and style length (Kato & Namai, 1987; Marathi & Jena, 2015; Virmani, 1994). To date, genes underlying four stigma length QTL in rice have been identified and shown to encode molecular functions related to gene transcription as well as hormone biosynthesis and signaling (Dang et al., 2022; Guo et al., 2022; Liu et al., 2015; Xu et al., 2019). Thanks to cereal genome research advances, these genes can now be assessed in a directed way, also in wheat (Yao et al., 2025). However, what genes control lodicule, carpel, and styloid development? How is stigma development and onset of receptivity coordinated up and down the spike and between florets?
3. *Enhanced pollen‐related traits*: Pollen abundance and duration of pollen shedding are prerequisites for successful fertilization and seed set of hybrids. So far, breeders phenotypically select lines with good anther extrusion or duration. Anther extrusion is a well‐documented multigenic trait; however, the underlying genes, other than *Rht*, remain to be identified. Pollen viability and amenability for wind dispersal need further assessment. An important consideration for this analysis will be to understand that pollen production cannot be selected for too strongly, as it may divert resources to male organs at the expense of yield. Developing approaches to effectively harvest, store, and apply pollen at the required time is another possibility.
4. *Gamete and organ interactions that drive fertilization (EPP*, *Figure* *4*): Pollination and fertilization each contribute to seed production, and further research that disentangles these processes will inform directed breeding or agronomic practices that target either process, either independently or in conjunction. Regarding organs, the interaction between stamens and pistils to coordinate optimum fertility would help determine the ideal timing for stigmas to be treated with airborne pollen. Pollen and pistils also undergo a complex interaction when pollen arrives on stigma hairs to promote pollen germination and then pollen tube growth, which is vital for fertilization.
5. *Climate effects on gamete development and fertility*: Gamete development, pollination, and fertilization are affected by variable climatic conditions. Seasonally distant events, such as warmer future winters, may affect the timing of gamete formation, while changes in irradiation and temperature will alter flowering itself. Climatic conditions are also expected to be more erratic, with wheat reproduction likely to suffer from drought or high air humidity and rain across different geographies (Pequeno et al., 2021). To optimize the sustainability of fertilization for self‐pollinating and cross‐pollinating seed production systems, it is vital that we understand more about the impacts of these environmental threats on gamete development, pollination, and fertilization. In conjunction with advances in identifying and phenotyping important traits mentioned above, the ability to incorporate both genetic and environmental data via crop growth models may also provide greater insights into critical environmental covariables important to maximize fertility under different agroclimatic conditions.
6. *Expanded genetic diversity*: Given the well‐described limitations of wheat's floral biology and its history as an inbred cereal, it is possible that elite genotypes may not contain the necessary genetic variation to enhance floral biology traits for efficient outcrossing. Such restrictions in genetic variation can now be directly informed through advances in wheat genomics (Yao et al., 2025) and the availability of pan‐genome sequences (Tiwari et al., 2024). Increasing the cross‐pollination capacity in wheat may also require floral architectures that are beyond the currently available germplasm. Rye, as a cross‐pollinating relative of wheat, for example, possesses desirable outcrossing floral traits. Unlike wheat, rye extrudes long anthers from the florets and sheds a high amount of pollen (Lundqvist, 1957). Rye anthers can grow to more than double the size of wheat anthers (Athwal & Kimber, 1970; Pickett, 1993), suggesting that anther size and timing of anther dehiscence are likely selected features for successful cross‐pollination in rye. For example, wheat‐rye 4R addition lines showed positive effects on anther size and pollen number (Nguyen et al., 2015). Identifying and adopting wheat genotypes with rye‐like floral characters may help improve male traits for higher seed set. Thus, there is a need to search for new beneficial diversity in floral characteristics by screening diversity panels, wild wheats, or mutant collections or creating novel variants through targeted modification of key floral genes using gene editing tools such as the CRISPR/Cas system.

The wheat breeding community is committed to supporting research in reproductive biology to overcome challenges of hybrid seed production, which will help transform breeding of this staple crop for improved food production. It is the aim of the wheat industry and wheat researchers to secure wheat pollination and fertilization under changing climatic conditions so that the benefits of inbred and hybrid wheat can be reaped by farmers and society. While sustaining self‐pollination of (parental) lines, a robust cross‐pollination success rate exceeding 75% of self‐pollination should be possible in an ever‐widening set of genetics and environments. Adequate investment from funding bodies and proactive public‐private partnerships are necessary to meet these goals.

## AUTHOR CONTRIBUTIONS

**Antje Rohde**: Conceptualization; data curation; formal analysis; writing—original draft; writing—review and editing. **Marc C. Albertsen**: Conceptualization; writing—review and editing. **Scott A. Boden**: Conceptualization; writing—original draft; writing—review and editing. **Pauline Bansept‐Basler**: Conceptualization; data curation; writing—review and editing. **Philipp H. G. Boeven**: Conceptualization; data curation; writing—review and editing. **Colin Cavanagh**: Conceptualization; data curation; writing—review and editing. **Laura E. Dixon**: Conceptualization; writing—review and editing. **Claus Frohberg**: Conceptualization; data curation; writing—review and editing. **Lucie Griffe**: Data curation; writing—review and editing. **Jacob Lage**: Conceptualization; data curation; writing—review and editing. **Leah Maeder**: Data curation; writing—review and editing. **Marina Millán‐Blánquez**: Conceptualization; writing—review and editing. **Paul D. Olson**: Conceptualization; writing—review and editing. **Laura Röhrig**: Data curation; writing—review and editing. **Thorsten Schnurbusch**: Conceptualization; writing—review and editing. **Cristóbal Uauy**: Writing—review and editing. **Ryan Whitford**: Conceptualization; data curation; writing—original draft; writing—review and editing.

## CONFLICT OF INTEREST STATEMENT

Ryan Whitford receives research support from ARC Industry Grant IM230100042 co‐funded by BASF. Antje Rohde and Jacob Lage are employed by KWS, Pauline Bansept‐Basler by Bayer, Philipp H. G. Boeven by Limagrain, Colin Cavanagh and Claus Frohberg by BASF, Lucie Griffe and Laura Röhrig by RAGT, Leah Maeder by Corteva, and Paul D. Olson by Syngenta.
